# Toxicity Profiles of Fractionated Radiotherapy, Contemporary Stereotactic Radiosurgery, and Transsphenoidal Surgery in Nonfunctioning Pituitary Macroadenomas

**DOI:** 10.3390/cancers11111658

**Published:** 2019-10-26

**Authors:** Chia-Lun Chang, Kevin Sheng-Po Yuan, Alexander T.H. Wu, Szu-Yuan Wu

**Affiliations:** 1 Department of Internal Medicine, School of Medicine, College of Medicine, Taipei Medical University, Taipei 106, Taiwan; richardch9@hotmail.com; 2 Department of Hemato-Oncology, Wan Fang Hospital, Taipei Medical University, Taipei 106, Taiwan; 3 Department of Otorhinolaryngology, Wan Fang Hospital, Taipei Medical University, Taipei 106, Taiwan; dryuank@gmail.com; 4 Ph.D. Program for Translational Medicine, Taipei Medical University, Taipei, Taiwan; chaw1211@tmu.edu.tw; 5 Department of Food Nutrition and Health Biotechnology, College of Medical and Health Science, Asia University, Taichung 413, Taiwan; 6 Division of Radiation Oncology, Lo-Hsu Medical Foundation, Lotung Poh-Ai Hospital, Yilan 265, Taiwan; 7 Department of Radiology, School of Medicine, College of Medicine, Taipei Medical University, Taipei 106, Taiwan

**Keywords:** nonfunctioning pituitary macroadenoma, transsphenoidal surgery, stereotactic radiosurgery, fractionated radiotherapy, toxicity, stroke

## Abstract

Background: Here, we compared the toxicity profiles of contemporary stereotactic radiosurgery (SRS), modern fractionated radiotherapy (FRT), and transsphenoidal surgery used to treat nonfunctioning pituitary macroadenomas. Methods: We included the data of patients with nonfunctioning pituitary macroadenomas. To compare treatment outcomes, the patients were categorized groups 1 (those receiving modern FRT), 2 (those receiving contemporary SRS), and 3 (those receiving transsphenoidal surgery). The multivariable Cox proportional hazards regression analysis was performed to yielded adjusted hazard ratios (aHRs) and their 95% CIs for local recurrence in groups 2 and 3 compared with group 1. Results: We included the data of 248 patients with nonfunctioning pituitary macroadenomas. The analytical results revealed no significant differences in second primary brain or head and neck cancer, hypopituitarism, or optic nerve injury between the three cohorts. The multivariable Cox proportional hazards regression analysis revealed that compared with group 1, the aHRs (95% CIs) for stroke risk in groups 2 and 3 were 0.37 (0.14–0.99) and 0.51 (0.31–0.84), respectively. Conclusion: Contemporary SRS and transsphenoidal surgery for nonfunctioning pituitary macroadenoma treatment have equivalent toxicity profiles. However, modern FRT for nonfunctioning pituitary macroadenoma treatment might considerably increase stroke risk.

## 1. Introduction

Approximately 30% of pituitary tumors are clinically nonfunctioning pituitary adenomas that do not secrete an excess amount of hormones [1,2,3]. Of these, tumors measuring >1 cm in size are called pituitary macroadenomas [1,2,3]. Nonfunctioning pituitary macroadenomas are difficult to recognize clinically until they are sufficiently large to cause symptoms due to mass effects [4]. They are often discovered incidentally during imaging procedures conducted for reasons other than pituitary symptoms or disease [1,2,3]. Transsphenoidal surgery is considered the standard treatment for nonfunctioning pituitary macroadenomas [5,6].

Over 2006 to 2015, improvements have been noted in contemporary radiotherapy (RT), fractionated RT (FRT) techniques (including intensity-modulated RT (IMRT) and volumetric modulated arc therapy (VMAT)), and stereotactic radiosurgery (SRS) techniques (including the use of modern linear accelerator (Linac)-based radiosurgical systems (CyberKnife (Accuray, Sunnyvale, CA, USA) or Novalis ExacTrac X-Ray 6D (Brainlab, Munich, Germany))). These are considered first-line treatment options for nonfunctioning pituitary macroadenomas [7]. Due to the improvements in treatment conformity and dose homogeneity index in modern FRT and SRS techniques, scattered radiation to normal tissues has been reduced and the radiation dose delivered to gross tumors increased [8,9,10,11,12,13]. However, the toxicity profiles of different treatment modalities for nonfunctioning pituitary macroadenomas remain unclear, including those of modern RT techniques. A primary concern is that using modern FRT or SRS might still increase the dose of scattered radiation delivered to normal tissues, thereby increasing the risk of second primary cancers, stroke, iatrogenic optic neuropathy, or hypopituitarism compared with transsphenoidal surgery [14,15,16,17,18,19]. However, evidence supporting this concern is unavailable.

Research has reported the toxicity profiles of conventional RT techniques used in adjuvant treatment of pituitary adenoma after surgery [14,15,16]. However, the toxicities of modern RT techniques or comparison of the toxicities of modern FRT, modern SRS, and transsphenoidal surgery as first-line treatments for nonfunctioning pituitary macroadenomas are yet to be reported. Therefore, the present study compared the toxicity profiles of modern SRS, modern FRT, and transsphenoidal surgery to recommend the best choice of therapeutic modality for nonfunctioning pituitary macroadenomas. In addition, we recommend that the risk/benefit ratio, including the risk of toxicity, should be carefully considered during the selection of a therapeutic modality for nonfunctioning pituitary macroadenomas.

## 2. Patients and Methods

We conducted a population-based cohort study using data from Taiwan National Health Insurance Research Database (NHIRD), linked to the Taiwan Cancer Registry (TCR). The TCR was established in 1979 and contains 97% of the cancer cases in Taiwan [20]. The NHIRD includes all medical claims data on disease diagnoses, procedures, drug prescriptions, demographics, and enrollment profiles of all beneficiaries [21]. NHIRD and TCR are linked by encrypted patient identifiers. By using data from the two databases, we selected patients who had received a diagnosis of a nonfunctioning pituitary macroadenoma over January 1, 2006, to December 31, 2015. The follow-up duration was from the index date to December 31, 2015. The index date was the date of RT in the FRT and SRS cohorts or the date of transsphenoidal surgery in the surgery cohort. Patients who received treatments >3 months after pituitary adenoma diagnosis were excluded. Our protocols were reviewed and approved by the Institutional Review Board of Taipei Medical University (TMU-JIRB 201712019). The TCR of the Collaboration Center of Health Information Application contains detailed cancer-related information regarding RT doses and techniques [22,23,24,25,26,27,28]. The diagnoses of selected patients were confirmed on the basis of the two databases, and patients who had received a new diagnosis of a nonfunctioning pituitary macroadenoma were confirmed to have no other cancers. The inclusion criteria were a diagnosis of a nonfunctioning pituitary macroadenoma and minimum adequate RT dose ≥45 Gy in the FRT cohort or minimum adequate SRS dose ≥14 Gy in one fraction in the SRS cohort. Adjuvant RT, including SRS or FRT, was allowed in the surgery cohort. The exclusion criteria were a history of cancer before nonfunctioning pituitary macroadenoma diagnosis, missing sex data, hypopituitarism, optic nerve injury, stroke before nonfunctioning pituitary macroadenoma diagnosis, irradiation history before diagnosis, unclear microadenomas or macroadenomas, and functioning pituitary adenomas with symptoms of hormone hypersecretion. In addition, we excluded patients who underwent therapy for >12 weeks after nonfunctioning pituitary macroadenoma diagnosis and did not receive modern RT techniques with IMRT or VMAT in the FRT cohort or did not receive RT with modern Linac-based radiosurgical systems (CyberKnife or Novalis ExacTrac X-Ray 6D) in the SRS cohort. Finally, we selected patients with nonfunctioning pituitary macroadenomas and categorized them into the following groups on the basis of treatment modality to compare their toxicities: group 1 (modern FRT cohort), group 2 (modern SRS cohort), and group 3 (transsphenoidal surgery cohort). The median total dose and fraction size of RT were 50.4 and 1.8 Gy, respectively, in group 1 and 18 Gy in one fraction, respectively, in group 2.

Comorbidities adjusted for as covariates were hypertension (HTN), atrial fibrillation (AF), hyperlipidemia, and diabetes mellitus (DM) with disease severity (i.e., diabetes complications severity index). Only comorbidities observed 6 months before the index date were included; comorbid conditions were identified and included according to the main International Classification of Diseases, Ninth Revision, Clinical Modification (ICD-9-CM) diagnosis codes for the first admission or more than two repeated main diagnosis codes for subsequent visits to the outpatient department.

After adjustments for confounders, the time-dependent Cox proportional hazards model was used to model the time from the index date to the date of occurrence of second primary brain or head and neck cancer, hypopituitarism, optic nerve injury, or stroke in patients undergoing the treatments. Multivariable Cox regression analysis was performed to calculate the hazard ratio (HR) for determining whether factors such as different therapies, age, sex, HTN, AF, hyperlipidemia, and DM are significant independent predictors. The independent predictors were controlled for in the analysis, and the endpoint was irradiation-related toxicity (such as second primary brain or head and neck cancer, hypopituitarism, optic nerve injury, and stroke) in the treatment groups, with group 1 (FRT) serving as the control arm. Second primary cancers were defined as cancers that may occur months or years after the original (primary) cancer was diagnosed and treated [29]. Thus, the definition of second primary brain or head and neck cancers in our study is brain cancers or head and neck cancers occurring months or years after nonfunctioning pituitary macroadenomas treated using FRT, SRS, or transsphenoidal surgery.

After adjustments for confounders, toxicity was estimated using time-dependent Cox proportional hazards model curves for second primary brain or head and neck cancer, hypopituitarism, optic nerve injury, and stroke in patients undergoing different treatments. After adjustments for confounders, the time-dependent Cox proportional hazards model was used to model the time from the index date to the date of occurrence of second primary brain or head and neck cancer, hypopituitarism, optic nerve injury, or stroke in patients undergoing the treatments. In the multivariable analysis, HRs were adjusted for age, sex, HTN, AF, hyperlipidemia, and DM and for different treatments. All analyses were performed using SAS software (version 9.3; SAS Institute, Cary, NC, USA). A two-tailed *p* of <0.05 was considered statistically significant.

Large randomized controlled trials (TMU-JIRB No. 201712019) with adequate sample sizes have yet to compare the toxicities of contemporary stereotactic radiosurgery (SRS), modern fractionated radiotherapy (FRT), and transsphenoidal surgery for the treatment of nonfunctioning pituitary macroadenomas. This study is the first to demonstrate that contemporary SRS and transsphenoidal surgery used for the treatment of nonfunctioning pituitary macroadenomas have equivalent toxicity profiles. Modern FRT for the treatment of nonfunctioning pituitary macroadenomas might be associated with a considerably high stroke risk.

## 3. Results

After reviewing 2006 to 2015 TCR data, we included the data of 248 patients with nonfunctioning pituitary macroadenomas in our study (Table 1). Overall, 133 patients received modern FRT, 53 modern SRS, and 62 transsphenoidal surgery. No significant differences in sex, HTN, AF, hyperlipidemia, or DM were observed between the three cohorts. However, patients who received FRT or SRS were older than those who received transsphenoidal surgery. The mean ages of the patients in the modern FRT, modern SRS, and transsphenoidal surgery cohorts were 52.7, 43.2, and 40.0 years, respectively, and their median follow-up durations were 4.1, 3.4, and 3.2 years, respectively. The age distribution (by 10-year age intervals) was balanced among the three groups (Table 1). No significant differences in second primary brain or head and neck cancer, hypopituitarism, or optic nerve injury were observed between the three treatment cohorts. However, we observed a significant difference in stroke incidence between the three treatment cohorts. The crude stroke incidence rates were 24.8%, 9.4%, and 13.3% in the FRT, SRS, and transsphenoidal surgery cohorts, respectively (Table 2). According to our multivariable Cox proportional hazards regression analysis of hypopituitarism risk in patients with nonfunctioning pituitary macroadenomas who received different treatments, treatment type was not a significant independent prognostic factor for hypopituitarism, second primary brain or head and neck cancer, or optic nerve injury (Table 3, Table 4 and Table 5). However, in our multivariable Cox proportional hazards regression analysis, compared with the FRT cohort, the adjusted hazard ratios (aHRs; 95% CIs) for stroke in the SRS and transsphenoidal surgery cohorts were 0.37 (0.14–0.99) and 0.51 (0.31–0.84), respectively (Table 6). Both our univariable and multivariable Cox proportional hazards regression analyses indicated that SRS was associated with the lowest stroke risk compared with the other treatments (Table 6). FRT was associated with a higher stroke risk than the other treatments. Compared with those aged 1 to 17 years, the aHRs (95% CIs) for stroke in patients aged 40 to 49, 50 to 59, 60 to 69, and ≥70 years were 1.42 (1.08–2.42), 1.91 (1.57–3.57), 2.69 (1.05–5.88), and 4.72 (1.74–6.81), respectively (Table 6).

Supplemental Materials Figures S1, S2, and S3 illustrate the Kaplan–Meier curves for cumulative stroke risk in the FRT, SRS, and transsphenoidal surgery cohorts, respectively. Stroke risk was higher with a nearly statistical trend in the FRT cohort than in those undergoing SRS, but it did not reach statistical significance (log-rank test, *p* = 0.058; Supplemental Materials Figure S1). Stroke risk was considerably higher in the FRT cohort than in the transsphenoidal surgery cohort (log-rank test, *p* = 0.005; Supplemental Materials Figure S2). The crude Kaplan–Meier curves for stroke risk were not significantly different between the SRS and transsphenoidal surgery cohorts (log-rank test, *p* = 0.485; Supplemental Materials Figure S3).

## 4. Discussion

Transsphenoidal surgery can be suggested as the first treatment choice for nonfunctioning pituitary macroadenomas [5,30], mainly because transsphenoidal surgery is associated with low rates of local recurrence (LR) and irradiation-related toxicities such as secondary brain or head and neck cancer, stroke, or optic nerve injury [17,19,31,32,33,34,35]. However, no study has clearly compared the toxicity profiles of FRT, SRS, and transsphenoidal surgery, all used for pituitary macroadenoma treatment. Thus, the present study is the first to conduct a long-term follow-up and compare the toxicity profiles of FRT, SRS, and transsphenoidal surgery used for pituitary macroadenoma treatment. Our findings could answer questions regarding whether modern RT techniques, including FRT and SRS, are more toxic than transsphenoidal surgery in treating pituitary macroadenomas. Relatively young patients prefer transsphenoidal surgery rather than RT because of late irradiation-related toxicities such as secondary brain or head and neck cancer, stroke, or optic nerve injury [17,19,31,32,33,34,35].

As presented in Table 1, the FRT and SRS cohorts included a higher number of older patients than did the transsphenoidal surgery cohort. Older patients might be more intolerant to anesthesia or surgery, and thus, might be more likely to be selected in the FRT and SRS cohorts [36]. However, despite old age being an independent risk factor for overall mortality and the SRS and FRT cohorts’ having a higher number of older patients, SRS was still associated with the lowest death risk, and no significant difference in mortality was observed between the FRT and transsphenoidal surgery cohorts in our previous study [7]. The three treatments could be ordered in terms of the LR rate of pituitary macroadenomas in the ascending order as SRS > FRT > transsphenoidal surgery [7]. SRS seemed to have the greatest effect in reducing LR and overall mortality [7]. In addition, the toxicity profiles, including second primary brain or head and neck cancer, hypopituitarism, and optic nerve pathway injury, seemed to have no significant difference when the SRS cohort was compared with the FRT and transsphenoidal surgery cohorts (Table 1). The only significant toxicity was higher stroke risk in the FRT cohort (Table 1). Moreover, we estimated the risk factors for stroke, including sex, age, HTN, and DM with different disease severity levels, hyperlipidemia, and AF, to estimate the true late toxicity of stroke. After adjustments for these covariates, FRT remained independently associated with stroke risk compared with SRS and transsphenoidal surgery (Table 6).

Studies are yet to evaluate the risk of iatrogenic hypopituitarism associated with FRT, SRS, or transsphenoidal surgery performed to treat nonfunctioning pituitary macroadenomas. As revealed in Table 1, FRT, SRS, or transsphenoidal surgery was associated with an approximately 10% iatrogenic hypopituitarism risk, corroborating the result of Kong et al. that SRS and FRT are associated with an 11.5% iatrogenic hypopituitarism risk [37]. Our Cox proportional hazards multivariate analysis revealed no significant differences in iatrogenic hypopituitarism risk after FRT, SRS, or transsphenoidal surgery (Table 3). Notably, patients aged ≥60 years had a lower iatrogenic hypopituitarism risk than did other patients (Table 3). These findings are potentially attributable to the competing risk of death; in particular, older patients had a higher risk of death before iatrogenic hypopituitarism than did younger patients, thus explaining the lower risk of iatrogenic hypopituitarism in such older patients [38]. A review of the literature revealed that the present study is the first and largest to examine the risk of iatrogenic hypopituitarism after FRT, SRS, and transsphenoidal surgery performed for nonfunctioning pituitary macroadenomas [37].

In our study, the risk of iatrogenic optic pathway injury was high after FRT, SRS, and transsphenoidal surgery. SRS and transsphenoidal surgery were associated with a nearly 20% iatrogenic optic pathway injury risk, whereas FRT was associated with a 35.3% iatrogenic optic pathway injury risk (Table 1). Although our multivariate analysis did not reveal significant differences in the risk of iatrogenic optic pathway injury associated with FRT, SRS, or transsphenoidal surgery (Table 4), the crude risk trend was higher in the FRT cohort (Table 2 and Table 4). Research estimating iatrogenic optic pathway injury risk after FRT, SRS, or transsphenoidal surgery for nonfunctioning pituitary macroadenoma treatment has been scant. Only one study reported the occurrence of radiation-induced optic neuropathy (13%) in 55 patients with pituitary adenoma who received fractionated stereotactic RT; however, this study did not report whether these were cases of pituitary microadenomas or macroadenomas [19]. The results of the present study, with a large sample size, can serve as a suitable reference for physicians when making treatment decisions regarding therapeutic modalities, in terms of their effects and toxicities, for patients with nonfunctioning pituitary macroadenomas [7].

Due to ill-defined radiation or other carcinogen–related second primary cancers, the sequelae and other etiologies related to brain or head and neck cancer after FRT, SRS, or transsphenoidal surgery could not be removed in our study. Moreover, even in the transsphenoidal surgery cohort, 17.7% of the patients without a history of radiation exposure had secondary brain or head and neck cancer (Table 1). Our Cox proportional hazards regression analysis revealed no significant differences in the risk of second primary brain or head and neck cancer between the patients in the three cohorts. We noted only one independent risk factor: age (Table 5). A younger age was associated with a greater risk of second primary brain or head and neck cancer. This finding might be due to the competing risk of death; specifically, older patients have a higher mortality rate and are thus more likely to die before the development of second primary brain or head and neck cancer [38]. Therefore, the greater number of younger patients in the transsphenoidal surgery cohort explains the non-significant difference in the risk of second primary brain or head and neck cancer [38]. Studies have revealed that both FRT and SRS used to treat pituitary adenoma could increase secondary brain cancer risk [31,32,33]. Compared with SRS, FRT may be associated with a higher secondary brain or head and neck cancer risk in our study, but the difference was non-significant in our multivariate analysis (Table 5). We included secondary head and neck cancer because modern therapeutic modalities, particularly FRT, can provide higher scattered radiation doses to the head and neck areas, instead of only being restricted to intracranial areas [18,39]. Moreover, scattered radiation might increase second primary head and neck cancer risk [18,40]. Our multivariate analysis did not reveal significant differences in the risk of second primary brain or head and neck cancer between FRT, SRS, and transsphenoidal surgery (Table 5). These findings might be a result of FRT involving a lower scattered radiation dose to the head and neck area than that found with therapeutic approaches in studies on head and neck cancer treatment [18,40].

Stroke incidence may be higher in patients with pituitary adenoma than in the general population [15]. Several case reports have described stroke development after RT for pituitary adenoma [14,16]. As presented in Table 1, the crude stroke risk was higher in the FRT cohort than in the SRS and transsphenoidal surgery cohorts. Our multivariate Cox regression analysis indicated that older age and FRT were independent risk factors for stroke in all three treatment cohorts (Table 6). Studies are yet to estimate stroke risk in different treatments for nonfunctioning pituitary macroadenomas. Hence, the present study is the first to demonstrate a clinically significant increase in stroke risk after FRT. Since there is no reported association between nonfunctioning pituitary macroadenomas and stroke risk, this significant risk is attributable to FRT. Before advising FRT for nonfunctioning pituitary macroadenoma treatment, physicians should carefully consider the corresponding risk/benefit ratio, including stroke risk. Notably, we observed that SRS was not associated with an increased stroke risk compared with transsphenoidal surgery (Table 6 and Supplemental Materials Figure S3). Studies have described RT for head and neck cancer as being associated with an increased stroke risk [17,35]. These findings are compatible with our results revealing that scattered radiation to vessels related to endothelial damage was associated with a high stroke risk [17,35,41]. Older age was also a significant independent risk factor for stroke in our study, and this result was consistent with the findings of previous studies (Table 6) [42,43]. However, no study has demonstrated that FRT for nonfunctioning pituitary macroadenoma treatment increases stroke risk compared with SRS or transsphenoidal surgery. In addition, no study has estimated stroke risk associated with FRT, SRS, and transsphenoidal surgery used for treating nonfunctioning pituitary macroadenomas. Only one study demonstrated that adjuvant RT after heterogeneous surgical procedures, including transsphenoidal surgery, craniotomy, and second surgical treatment, did not increase stroke risk compared with surgery alone for nonfunctioning or functional pituitary adenoma treatment; however, the study did not mention whether microadenomas or macroadenomas were included [15]. The relative stroke risk in patients treated with adjuvant RT was not significantly different from that in patients who received surgery alone [15]. Our findings revealed that FRT used for pituitary adenoma treatment might increase stroke risk compared with SRS and transsphenoidal surgery. In addition, our findings demonstrate that SRS was relatively safe and effective and had less iatrogenic toxicity in the treatment of pituitary adenoma compared with FRT (Table 6 and Supplemental Materials Figures S1–S3).

In our study, the FRT cohort received standard radiation dose fractionation for the treatment of nonfunctioning pituitary macroadenomas. The development of radiation-induced central nervous system (CNS) toxicity is linked to the total dose, dose–fractionation relationship, and volume effect [44,45]. In theory, a standard radiation dose causes less CNS or vessel toxicity than do hypofractionated regimens [46]. Repeated small radiation doses are less damaging to cells than an equivalent total dose administered in a large fraction [47]. Late-reacting tissues, exemplified by the normal CNS, have a low α/β ratio, typically 1 to 3 Gy (range 0.5–6 Gy) [48]. The slow growth and presumed limited dividing capacity of benign tumors such as pituitary adenoma have led to the assumption these tumors have a low α/β ratio, but this remains unconfirmed. Late-responding tissues are more spared by fractionation than are early-responding tissues; this is the principle of FRT, particularly as applied to the CNS, which has an α/β ratio of 1 to 2 Gy, where fractionation preferentially spares normal brain compared with a high α/β ratio tumor [49]. Owing to numerous nerves and vessels surrounding the pituitary, physicians in Taiwan use FRT with standard radiation dose instead of a hypofractionated regimen for treatment of pituitary adenoma (Table 1). Thus, most patients received standard fractions in the FRT cohort with median total dose and fraction of FRT of 50.4 and 1.8 Gy, respectively (Table 1). However, even with the improvements in treatment conformity and dose homogeneity index in modern FRT [8,9,10,11], stroke risk after FRT remains higher than that after SRS or transsphenoidal surgery (Table 6). The results were still compatible with those of previous studies on conventional RT techniques used in adjuvant treatment of pituitary adenoma after surgery [14,15,16].

The strength of our study was that it was the first to compare the toxicity profiles of modern SRS, modern FRT, and transsphenoidal surgery, which could serve as a reference in selecting the optimal therapy for treating nonfunctioning pituitary macroadenomas. This is crucial because before selecting a therapeutic modality for treating nonfunctioning pituitary macroadenomas, physicians should carefully consider the risk/benefit ratio, including the risk of toxicity. Furthermore, among relevant studies, the present study has the largest sample size and provides the highest curative therapeutic consistency in modern RT techniques or surgical procedures for nonfunctioning pituitary macroadenoma treatment. Modern SRS and transsphenoidal surgery were determined to have lower iatrogenic toxicity levels than modern FRT for treating nonfunctioning pituitary macroadenomas. Our outcomes indicate that even modern FRT for the treatment of nonfunctioning pituitary macroadenomas was associated with a higher stroke risk than modern SRS and transsphenoidal surgery (Table 6). These findings could also be considered in future clinical practice and randomized controlled studies.

This study has some limitations. First, because all patients with nonfunctioning pituitary macroadenomas were enrolled from an Asian population, the corresponding ethnic susceptibility remains unclear; hence, caution should be exercised when extrapolating our results to non-Asian populations. Second, the diagnoses of all comorbid conditions were based on ICD-9-CM codes. Nevertheless, the Taiwan National Health Insurance Administration randomly reviews charts and interviews patients to verify the accuracy of diagnoses, and hospitals with outlier charges or practices may be audited and subsequently be heavily penalized if malpractice or discrepancies are identified. Third, to prevent the creation of several subgroups, various adjuvant treatments after transsphenoidal surgery were not categorized separately during the analyses. Thus, the toxicities of different adjuvant treatments remain unclear. Therefore, even when SRS or FRT was applied after transsphenoidal surgery in this study, the toxicity profiles observed for the transsphenoidal surgery cohort remained lower than those observed for the FRT cohort. These conclusions remained unchanged even after we added subgroups for adjuvant treatment. Fourth, in the used database, treatment plans were unavailable. To date, no published studies have compared the differences of margin prescription and the dose delivered to the organ at risk between FRT and SRS. According to previous studies, SRS techniques, which include modern Linac-based radiosurgical systems (CyberKnife or Novalis ExacTrac X-Ray 6D), seemed to lead to greater improvements in treatment conformity and dose homogeneity index than FRT in different studies [8,9,10,11,12,13]; in addition, in SRS, scattered radiation to brain tissues or peripheral vessels is less, and the radiation dose delivered to gross tumors is greater. Accordingly, to obtain crucial information on population specificity and disease occurrence, a large-scale randomized trial comparing carefully selected patients undergoing suitable treatments is essential. Finally, the TCR does not contain information regarding dietary habits, socioeconomic status, or body mass index, all of which may be mortality risk factors. However, considering the magnitude and statistical significance of the observed effects in this study, these limitations are unlikely to affect the conclusions.

## 5. Conclusions

In nonfunctioning pituitary macroadenoma treatment, SRS and transsphenoidal surgery have equivalent toxicity profiles, whereas modern FRT may considerably increase stroke risk.

## Figures and Tables

**Table 1:** Characteristics of patients with nonfunctioning pituitary macroadenomas who received fractionated radiotherapy, stereotactic radiosurgery, or transsphenoidal Surgery.

Variables	Fractionated Radiotherapy (*n* = 133)	Stereotactic Radiosurgery (*n* = 53)	Transsphenoidal Surgery (*n* = 62)	*p* Value
	*n* (%)	*n* (%)	*n* (%)	
Sex				0.877
Male	79 (59.4)	32 (60.4)	208 (57.5)	
Female	54 (40.6)	21 (39.6)	154 (42.5)	
Age (y)				0.024
1–17	22 (16.5)	9 (17.0)	96 (26.5)	
18–29 30–39 40–49 50–59 60–69 ≥70	11 (8.3) 26 (19.5) 24 (18.0) 17 (12.8) 19 (14.3) 14 (10.5)	7 (13.2) 12 (22.6) 8 (15.1) 6 (11.3) 3 (5.7) 8 (15.1)	48 (13.3) 46 (12.7) 67 (18.5) 50 (13.8) 35 (9.7) 20 (5.5)	
Hypertension				0.111
No	30 (22.6)	19 (35.8)	84 (23.2)	
Yes	17 (12.8)	6 (11.3)	69 (19.1)	
Diabetes				0.583
No	23 (17.3)	10 (18.9)	84 (23.2)	
DSCI = 1	59 (44.4)	26 (49.1)	153 (42.3)	
DSCI > 1	51 (38.3)	17 (32.1)	125 (34.5)	
Hyperlipidemia				0.029
No	93 (69.9)	38 (71.7)	286 (79.0)	
Yes	34 (25.6)	9 (17.0)	51 (14.1)	
Atrial fibrillation				0.868
No	84 (63.2)	32 (60.4)	232 (64.1)	
Yes	49 (36.8)	21 (39.6)	130 (35.9)	
Radiation dose (median, Gy)	50.4	18	0	<0.001

DCSI, diabetes complications severity index.

**Table 2:** Toxicity profiles of patients with nonfunctioning pituitary macroadenomas who received fractionated radiotherapy, stereotactic radiosurgery, or transsphenoidal surgery.

Variables	Fractionated Radiotherapy (*n* = 133)	Stereotactic Radiosurgery (*n* = 53)	Transsphenoidal Surgery (*n* = 62)	*p* Value
	*n* (%)	*n* (%)	*n* (%)	
Second primary brain or head and neck cancer	26 (19.5)	7 (13.2)	64 (17.7)	0.593
Stroke	33 (24.8)	5 (9.4)	48 (13.3)	0.003
Hypopituitarism	14 (10.5)	5 (9.4)	37 (10.2)	0.976
Optic nerve pathway injury	47 (35.3)	12 (22.6)	95 (26.2)	0.089

**Table 3:** Cox proportional hazards regression analysis of hypopituitarism risk in patients with nonfunctioning pituitary macroadenomas who received different treatments.

Variables	Crude HR (95% CI)		Adjusted HR * (95% CI)		*p* Value
Therapeutic modality (reference, fractionated radiotherapy)					
Stereotactic radiosurgery	0.839	(0.30, 2.34)	0.780	(0.27, 2.25)	0.6457
Transsphenoidal surgery	0.915	(0.49, 1.70)	0.863	(0.45, 1.66)	0.6579
Age (y; reference, 1–17 y)					
18–29	1.591	(0.77, 3.31)	1.751	(0.82, 3.75)	0.1499
30–39	1.466	(0.73, 2.97)	1.543	(0.74, 3.23)	0.2485
40–49	0.609	(0.24, 1.56)	0.500	(0.19, 1.31)	0.1569
50–59	0.922	(0.34, 2.53)	0.664	(0.23, 1.95)	0.4572
60–69	0.198	(0.03, 1.49)	0.191	(0.11, 0.74)	0.0247
≥70	0.177	(0.41, 1.87)	0.169	(0.12, 0.60)	0.0296
Sex (reference, female)					
Male	1.026	(0.46, 2.27)	1.263	(0.52, 3.04)	0.6030
Diabetes (reference, no diabetes)					
DSCI = 1	1.645	(0.71, 3.82)	1.744	(0.73, 4.19)	0.2135
DSCI > 1	2.069	(0.89, 4.79)	2.765	(0.73, 6.75)	0.1256
Hyperlipidemia (reference, no hyperlipidemia)					
Yes	1.245	(0.54, 2.88)	1.529	(0.64, 3.64)	0.3377
Hypertension (reference, no hypertension)					
Yes	1.697	(0.55, 5.29)	2.597	(0.79, 8.53)	0.1157
Atrial fibrillation (reference, no atrial fibrillation)					
Yes	2.13	(1.94, 3.35)	2.113	(0.95, 3.25)	0.4361

* All the aforementioned variables were used in the multivariate analysis. DCSI, diabetes complications severity index; HR, hazard ratio.

**Table 4:** Cox proportional hazards regression analysis of optic nerve injury risk in patients with nonfunctioning pituitary macroadenomas who received different treatments.

Variables	Crude HR (95% CI)		Adjusted HR * (95% CI)		*p* Value
Therapeutic modality (reference, fractionated radiotherapy)					
Stereotactic radiosurgery	0.637	(0.34, 1.21)	0.580	(0.29, 1.15)	0.1176
Transsphenoidal surgery	0.956	(0.67, 1.36)	0.987	(0.68, 1.43)	0.9432
Age (y; reference, 1–17 y)					
18–29	0.615	(0.34, 1.11)	0.552	(0.30, 1.01)	0.3081
30–39	0.696	(0.43, 1.12)	0.666	(0.40, 1.12)	0.1153
40–49	0.726	(0.44, 1.20)	0.604	(0.35, 1.03)	0.1034
50–59	1.007	(0.58, 1.76)	1.014	(0.56, 1.82)	0.1824
60–69	1.987	(1.15, 3.42)	1.561	(0.83, 2.93)	0.2928
≥70	0.877	(0.41, 1.87)	0.669	(0.28, 1.60)	0.1596
Sex (reference, female)					
Male	1.276	(0.79, 2.07)	1.003	(0.59, 1.7)	0.9924
Diabetes (reference, no diabetes)					
DSCI = 1	1.152	(0.76, 1.75)	1.744	(0.73, 4.19)	0.2135
DSCI > 1	1.311	(0.61, 2.83)	1.321	(0.56, 3.14)	0.5284
Hyperlipidemia (reference, no hyperlipidemia)					
Yes	1.043	(0.62, 1.77)	0.883	(0.51, 1.52)	0.6536
Hypertension (reference, no hypertension)					
Yes	1.359	(0.70, 2.62)	1.180	(0.60, 2.33)	0.6322
Atrial fibrillation (reference, no atrial fibrillation)					
Yes	1.244	(0.89, 1.74)	1.181	(0.76, 1.83)	0.4558

* All the aforementioned variables were used in the multivariate analysis. DCSI, diabetes complications severity index; HR, hazard ratio.

**Table 5:** Cox proportional hazards regression analysis of second primary brain or head and neck cancer risk in patients with nonfunctioning pituitary macroadenomas who received different treatments.

Variables	Crude HR (95% CI)		Adjusted HR * (95% CI)		*p* Value
Therapeutic modality (reference, fractionated radiotherapy)					
Stereotactic radiosurgery	0.57	(0.25, 1.32)	0.532	(0.23, 1.26)	0.1495
Transsphenoidal surgery	0.971	(0.62, 1.53)	0.909	(0.56, 1.48)	0.7005
Age (y; reference, 1–17 y)					
18–29	0.745	(0.22, 0.91)	0.658	(0.17, 0.76)	0.0074
30–39	0.746	(0.24, 0.84)	0.518	(0.22, 0.81)	0.0101
40–49	0.663	(0.24, 0.88)	0.511	(0.21, 0.80)	0.0085
50–59	0.562	(0.47, 1.59)	0.479	(0.40, 1.50)	0.0461
60–69	0.434	(0.18, 1.04)	0.304	(0.07, 0.58)	0.0028
≥70	0.375	(0.24, 1.38)	0.211	(0.11, 0.86)	0.0238
Sex (reference, female)					
Male	1.087	(0.6, 1.96)	1.292	(0.66, 2.53)	0.4547
Diabetes (reference, no diabetes)					
DSCI = 1	1.318	(0.79, 2.20)	1.304	(0.76, 2.23)	0.3315
DSCI > 1	1.661	(0.49, 1.72)	1.512	(0.50, 1.66)	0.7633
Hyperlipidemia (reference, no hyperlipidemia)					
Yes	1.047	(0.54, 2.03)	1.009	(0.51, 2.00)	0.9796
Hypertension (reference, no hypertension)					
Yes	1.088	(0.58, 4.05)	1.961	(0.41, 6.28)	0.2047
Atrial fibrillation (reference, no atrial fibrillation)					
Yes	1.104	(0.73, 1.68)	1.300	(0.76, 2.22)	0.3382

* All the aforementioned variables were used in the multivariate analysis. DCSI, diabetes complications severity index; HR, hazard ratio.

**Table 6:** Cox proportional hazards regression analysis of stroke risk in patients with nonfunctioning pituitary macroadenomas who received different treatments.

Variables	Crude HR (95% CI)		Adjusted HR * (95% CI)		*p* Value
Therapeutic modality (reference, fractionated radiotherapy)					
Stereotactic radiosurgery	0.388	(0.15, 1)	0.371	(0.14, 0.89)	0.0387
Transsphenoidal surgery	0.530	(0.34, 0.83)	0.509	(0.31, 0.83)	0.0072
Age (y; reference, 1–17 y)					
18–29	1.359	(0.54, 3.45)	1.574	(0.61, 4.05)	0.3470
30–39	1.304	(0.54, 3.14)	1.267	(0.51, 3.13)	0.6077
40–49	1.523	(1.14, 3.57)	1.419	(1.08, 2.42)	0.0318
50–59	1.673	(1.13, 4.26)	1.906	(1.57, 3.57)	<0.0001
60–69	2.907	(1.71, 6.93)	2.686	(1.05, 5.88)	0.0395
≥70	5.546	(2.79, 7.34)	4.721	(1.74, 6.81)	0.0023
Sex (reference, female)					
Male	2.047	(1.19, 3.51)	1.312	(0.72, 2.41)	0.3796
Diabetes (reference, no diabetes)					
DSCI = 1	1.360	(0.69, 2.67)	1.554	(0.77, 3.13)	0.2181
DSCI > 1	1.942	(0.92, 4.12)	1.971	(0.91, 4.29)	0.0877
Hyperlipidemia (reference, no hyperlipidemia)					
Yes	2.707	(1.16, 6.30)	2.369	(0.96, 5.87)	0.0626
Hypertension (reference, no hypertension)					
Yes	2.197	(1.43, 3.38)	1.261	(0.72, 2.20)	0.4146
Atrial fibrillation (reference, no atrial fibrillation)					
Yes	1.300	(0.73, 2.31)	1.189	(0.64, 2.20)	0.5820

* All the aforementioned variables were used in the multivariate.

## Supplementary Materials

The following are available online at https://www.mdpi.com/2072-6694/11/11/1658/s1, Figure S1: Kaplan–Meier curves for stroke risk in patients with nonfunctioning pituitary macroadenomas who received stereotactic radiosurgery and those who received fractionated radiotherapy. Figure S2 Kaplan–Meier curves for stroke risk in patients with nonfunctioning pituitary macroadenomas who received transsphenoidal surgery and those who received fractionated radiotherapy. Figure S3 Kaplan–Meier curves for stroke risk in patients with nonfunctioning pituitary macroadenomas who received transsphenoidal surgery and those who received stereotactic radiosurgery.

## Abbreviations

aHR	adjusted hazard ratio
FRT	fractionated radiotherapy
HR	hazard ratio
ICD-9-CM	International Classification of Diseases, Ninth Revision, Clinical Modification
LR	local recurrence
