# Supplementary material for: Toxicity Profiles of Fractionated Radiotherapy, Contemporary Stereotactic Radiosurgery, and Transsphenoidal Surgery in Nonfunctioning Pituitary Macroadenomas
Source: Cancers (Basel). 2019 Oct 26;11(11):1658. doi: 10.3390/cancers11111658 (PMC6896065; doi:10.3390/cancers11111658)
Supplement: Supplementary file 1 [file cancers-11-01658-s001.pdf]

Supplementary Materials: Toxicity Profiles of Fractionated Radiotherapy, Contemporary Stereotactic Radiosurgery, and Transsphenoidal Surgery in Nonfunctioning Pituitary Macroadenomas

Chia-Lun Chang, Kevin Sheng-Po Yuan, Alexander T.H. Wu and Szu-Yuan Wu

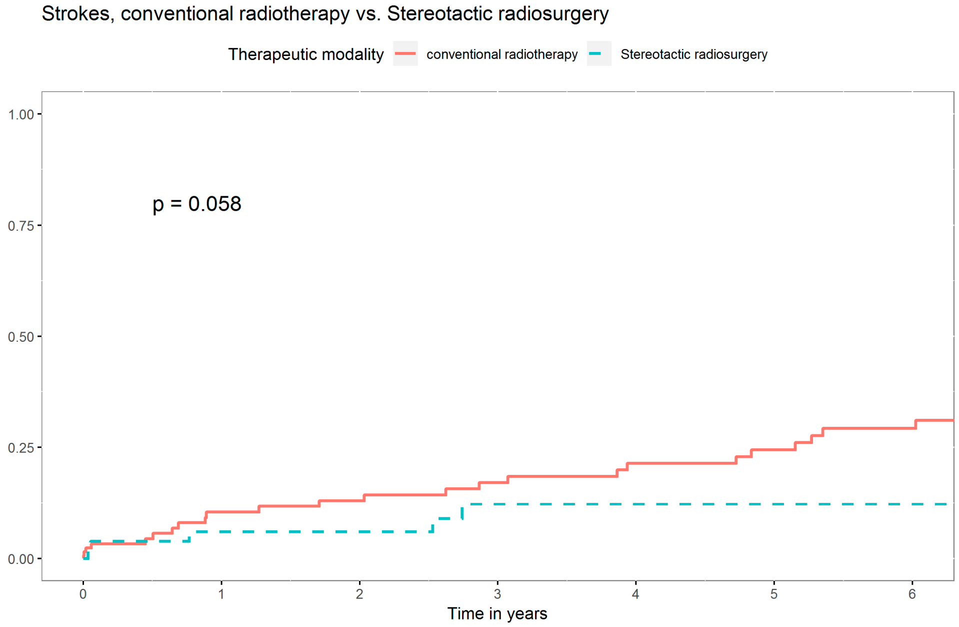

Figure S1. Kaplan–Meier curves for stroke risk in patients with nonfunctioning pituitary macroadenomas who received stereotactic radiosurgery and those who received fractionated radiotherapy.

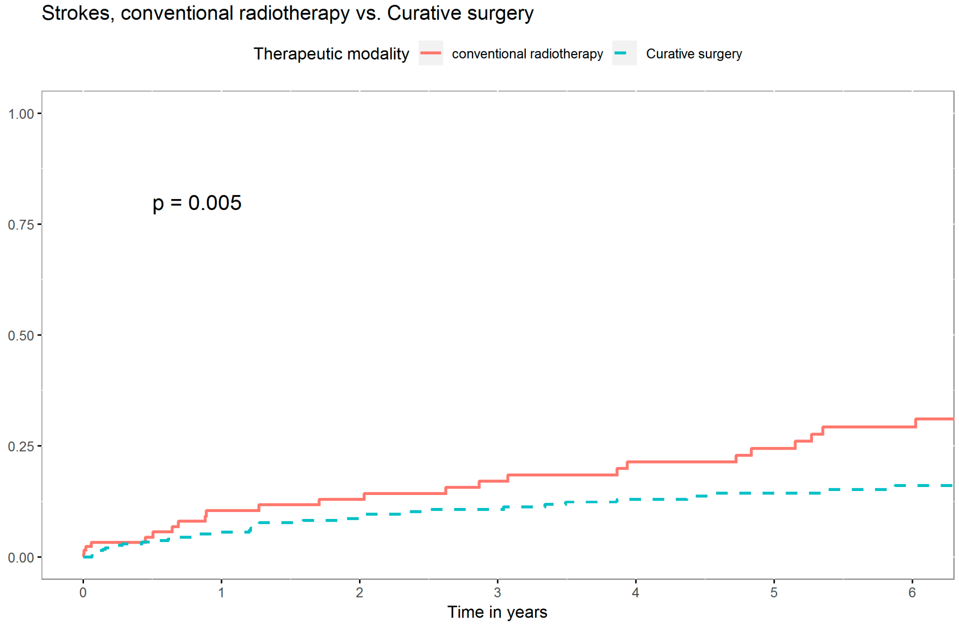

Figure S2. Kaplan–Meier curves for stroke risk in patients with nonfunctioning pituitary macroadenomas who received transsphenoidal surgery and those who received fractionated radiotherapy.

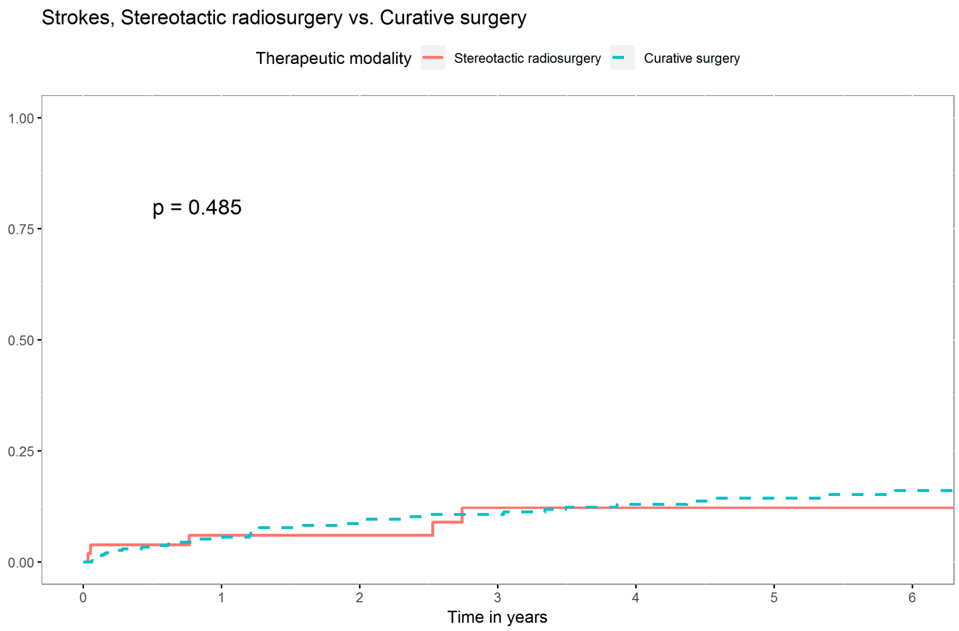

Figure S3. Kaplan–Meier curves for stroke risk in patients with nonfunctioning pituitary macroadenomas who received transsphenoidal surgery and those who received stereotactic radiosurgery.

© 2019 by the authors. Licensee MDPI, Basel, Switzerland. This article is an open access article distributed under the terms and conditions of the Creative Commons Attribution (CC BY) license (http://creativecommons.org/licenses/by/4.0/).
